# Reactive Oxygen Species-Mediated Mechanisms of Action of Targeted Cancer Therapy

**DOI:** 10.1155/2017/1485283

**Published:** 2017-06-18

**Authors:** Hanna-Riikka Teppo, Ylermi Soini, Peeter Karihtala

**Affiliations:** ^1^Department of Pathology, Medical Research Center Oulu, Oulu University Hospital and University of Oulu, Oulu, Finland; ^2^Department of Oncology and Radiotherapy, Medical Research Center Oulu, Oulu University Hospital and University of Oulu, Oulu, Finland

## Abstract

Targeted cancer therapies, involving tyrosine kinase inhibitors and monoclonal antibodies, for example, have recently led to substantial prolongation of survival in many metastatic cancers. Compared with traditional chemotherapy and radiotherapy, where reactive oxygen species (ROS) have been directly linked to the mediation of cytotoxic effects and adverse events, the field of oxidative stress regulation is still emerging in targeted cancer therapies. Here, we provide a comprehensive review regarding the current evidence of ROS-mediated effects of antibodies and tyrosine kinase inhibitors, use of which has been indicated in the treatment of solid malignancies and lymphomas. It can be concluded that there is rapidly emerging evidence of ROS-mediated effects of some of these compounds, which is also relevant in the context of drug resistance and how to overcome it.

## 1. Introduction

There has been significant progress in the development of novel oncological treatments during the last decade. However, radiotherapy and traditional chemotherapy still form the backbone of treatment in most malignancies. The importance of conventional cytotoxic chemotherapy is underlined in adjuvant treatments, where only trastuzumab and imatinib are currently approved in HER2- (human epidermal growth factor receptor 2-) positive early breast cancer and Kit-positive gastrointestinal stromal tumour (GIST) treatments, respectively. In metastatic disease, nevertheless, targeted cancer therapy has prolonged survival significantly. This has been observed especially in HER2-overexpressing breast cancer, renal cell carcinoma (RCC), GISTs, melanoma, and colorectal cancer (CRC) [1–5]. In several metastatic cancer trials, there have been plateaus in survival curves in patients treated with targeted therapies, even after relatively long follow-up periods. The current paradigm still suggests that metastatic cancer is curable extremely rarely and that drug resistance ultimately develops [6].

Reactive oxygen species (ROS) are a set of highly reactive molecules comprising singlet oxygen (^1^O_2_), superoxide (O_2_^•−^), hydroxyl radical (OH^•^), and hydrogen peroxide (H_2_O_2_). They have crucial roles in both physiological functions and tumour development [7]. Production of ROS is elevated in malignant compared with benign tissues as a result, for example, of increased metabolic rate, oncogene activation, and defective vasculature, leading to hypoxic areas, but several lines of evidence suggest that cancer tissues may upregulate levels of antioxidant enzymes to counteract increased oxidative stress, as reviewed elsewhere [8–10]. Excess ROS are quenched by enzymatic antioxidants such as superoxide dismutase (SOD), catalase, glutathione (GSH), and peroxiredoxins (Prxs) and nonenzymatic antioxidants such as vitamins E and A [8]. In addition, enzymes such as thioredoxin-1 (Trx-1) and GSH are able to restore REDOX-sensitive proteins to their proper function by reducing the cysteine residues within these proteins [10]. The expression of antioxidant proteins is controlled by the major antioxidant response regulator nuclear factor erythroid 2-related factor 2 (NFE2L2). NFE2L2 is activated during oxidative and electrophilic stress and released from its inhibitory molecule Kelch-like ECH-associated protein 1 regulator (Keap1) [10].

The effectiveness of traditional cancer chemotherapy is largely based on the generation of ROS and consequently on the increase of oxidative stress that exceeds the reduction capacity of cancerous tissue, leading ultimately to apoptosis or necrosis [10]. Also, many adverse effects of chemotherapy are due to excess ROS production in healthy tissues, such as anthracycline-mediated cardiotoxicity, and nephrotoxicity triggered by platinum compounds [11, 12]. Up to 50% of patients with cancer receive radiotherapy at some stage of their illness [13]. Both therapeutic and side effects of ionizing radiation during radiotherapy are mainly based on the interaction of OH^•^ with target tissue DNA [14].

Targeted cancer therapy mostly involves monoclonal antibodies, small-molecule tyrosine kinase inhibitors (TKIs) and, more recently, immunotherapies. In a broad context, some hormonal therapies such as tamoxifen therapy [15] can also be included in this category, but they are not discussed in this review. The rationale for targeted cancer therapies is to specifically disrupt certain upregulated pathways in malignant cells. Theoretically, this could lead to more effective cancer cell death, with less harmful effects. However, the compounds concerned have drug-specific and sometimes life-threatening adverse effects, and therefore, combinations of these treatments are often limited in a clinical setting [16]. At first, targeted cancer therapies were considered to be promising magic bullets with single targets [17, 18], but their wider clinical use has produced much information about their diverse mechanisms of action and development of drug resistance, where also ROS could have a substantial role.

In this paper, we will review the current evidence of ROS-mediated effects of antibodies and tyrosine kinase inhibitors that have European Medicines Agency (EMA) approval as regards the treatment of solid malignancies or lymphomas. Originally, we also aimed to address oncoimmunological compounds, but the ROS-mediated effects of these compounds are still largely unknown. Since most of the drugs discussed in this review are novel, the research field has so far been uncoordinated and somewhat sporadic. However, regarding some of the compounds concerned, there is rapidly emerging evidence of ROS-mediated effects and adverse effects. As far as we know, no previous reviews on this topic exist.

## 2. Tyrosine Kinase Inhibitors

Tyrosine kinase inhibitors are compounds of small molecular weight. Their small size enables oral administration of the drugs and effective penetrance through cell membranes, after which they exert their functions intracellularly. The main mechanism of action of all TKIs is competitive adenosine triphosphate inhibition at the catalytic binding site of tyrosine kinase, but TKIs differ considerably in their specificity against different kinases [19]. There are currently nearly 20 EMA-approved TKIs for the treatment of solid tumours or lymphomas, and the list is expanding rapidly (http://ema.europa.eu).

### 2.1. Sunitinib, Pazopanib, and Sorafenib

**Figure 1:**
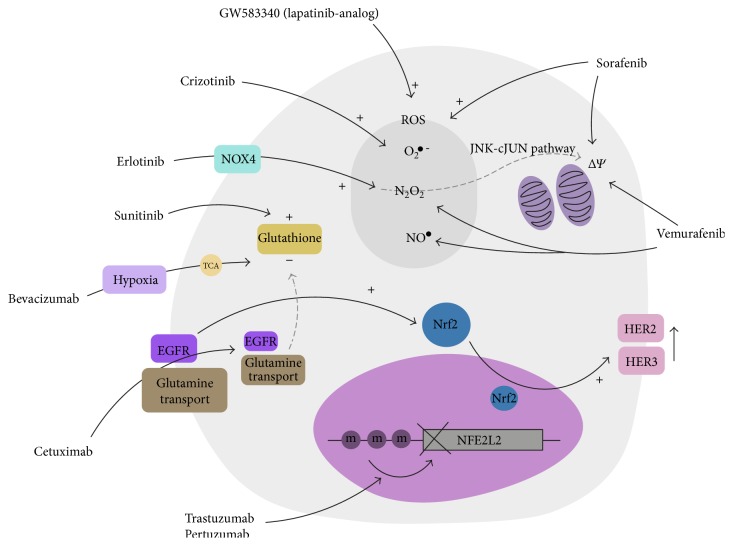
Expected ROS-mediated effects of targeted therapies. In addition to the respective signalling pathway that a targeted agent affects, the diagram represents their putative ROS-related effects. CRC: colorectal cancer; GISTs: gastrointestinal stromal tumours; RCC: renal cell carcinoma; ROS: reactive oxygen species; O_2_^•−^: superoxide; H_2_O_2_: hydrogen peroxide; NO^•^: nitric oxide; Δ*Ψ*: mitochondrial membrane polarity; TCA: tricarboxylic acid cycle; m: methylation; NFE2L2: nuclear factor erythroid 2-related factor 2.

Sunitinib was the first TKI approved for the treatment of advanced/metastatic RCC (in 2006), and currently, there are also indications for its use in the treatment of imatinib-resistant GISTs and pancreatic neuroendocrine carcinomas. Sunitinib is one of the most commonly administered TKIs in clinical practice. The main mechanism of action is mediated by effective blocking of vascular endothelial growth factor receptors (VEGFRs) 1–3, platelet-derived growth factor receptor-*α* (PDGFR-*α*) and PDGFR-*β*, and c-Kit [20]. Possible ROS-mediated cytotoxic effects of sunitinib have been evaluated, but results consistently demonstrate that there is no connection between ROS and cytotoxic effects, nor an association with sunitinib-induced vasoconstriction [21–23]. Indeed, there is evidence that sunitinib may even act as an antioxidant by ameliorating lipid peroxidation and increasing GSH levels in cisplatin-treated mice (Figure 1()). This not only reduces oxidative stress-triggered side effects but also improves chemotherapy efficacy [24] (Table 1). Sunitinib also inhibits the activity and expression of neuronal nitric oxide synthase (NOS), which has been associated with the neuroprotective effects of sunitinib in vitro and also with reduced vasodilation in animal experiments [25, 26]. Sunitinib combined with chloroquine increases inducible NOS, leading to an increase in reactive nitrogen species and apoptosis while, on the other hand, the increased level of GSH abrogates apoptosis [27]. Whether these effects are relevant as regards tumour growth remains unclear on the basis of the current literature. Interestingly, sunitinib induces incomplete autophagy in bladder-cancer cell lines but does not induce mitochondrial depolarization or the induction of ROS and rather targets lysosomes and induces lysosome-dependent cell death [22].

Pazopanib is also a wide-range TKI, which blocks not only VEGFRs, PDGFRs, and c-Kit, like sunitinib, but also targets fibroblast growth factor receptors-1 (FGFRs-1) and FGFRs-3, IL-2-inducible T-cell kinase, lymphocyte-specific protein tyrosine kinase, and macrophage colony-stimulating factor 1 receptor (c-Fms). Pazopanib is less well studied as regards the topic of this review, but at least pazopanib-triggered erythrocyte apoptosis has been suggested to be dependent on oxidative stress [28]. Another TKI, axitinib, has been indicated for the treatment of RCC. Besides its VEGFR-targeted effects, it also induces oxidative DNA damage, leading to mitotic catastrophe and a cellular senescence program. This further promotes natural killer (NK) cell-mediated recognition and elimination of RCC through the regulation NK-activating ligand expression [29]. This data has linked axitinib-mediated oxidative stress to its immunomodulatory effects, which is especially relevant in RCC, one of the most immunogenic cancers [30].

Sorafenib is multikinase inhibitor that blocks VEGFR-2 and VEGFR-3, PDGFR-*β*, and RAF-kinases, for example, consequently inhibiting proliferation and angiogenesis. Sorafenib is currently indicated for the treatment of metastatic RCC, hepatocellular carcinoma (HCC), and radioiodine refractory, differentiated thyroid cancer. Interestingly, the effectiveness of sorafenib is widely reliant on kinase-independent, ROS-mediated mechanisms, especially in mitochondria. In HCC HepG2 cell lines, sorafenib induces a significant increase in the production of ROS, mainly in mitochondria, which is followed by a rapid and deep depletion of GSH in the mitochondria, cytoplasm, and nuclei [31]. Coriat et al. demonstrated later that sorafenib particularly induces the production of H_2_O_2_, O_2_^•−^, and nitric oxide in HepG2 cells [32]. Superoxide dismutase (SOD) mimics efficiently inhibited the antiproliferative and cytotoxic effects of sorafenib and increased tumour growth in mice, while H_2_O_2_ and NO^•^ inhibitors had no effect on sorafenib cytotoxicity. Intriguingly, the authors consequently confirmed, in the series of HCC patients, that higher serum levels of advanced oxidation production proteins were predictive of prolonged relapse-free survival and overall survival. In another recent paper, it was suggested that sorafenib brought about elevated ROS production and inhibited mitochondrial respiration, and gene expression profiling also revealed that sorafenib led to a substantial change toward aerobic glycolysis [33]. In line with this, glucose withdrawal or glycolytic inhibitor dramatically improved sorafenib cytotoxicity. It thus appears that mitochondrial dysfunction and O_2_^•−^ production play a significant role in sorafenib treatment, facts that allow possible predictive biomarkers and combination treatments to be assessed in future clinical trials. Indeed, in a recent article, sorafenib was shown to disrupt the mitochondrial membrane potential in RCC, leading to increased ROS and thus breaking resistance to TRAIL-induced apoptosis [34]. Some compounds such as melatonin may increase the therapeutic action of sorafenib and induce further ROS increase during administration of both compounds [35].

### 2.2. Crizotinib

Recent landmark trials showed a huge improvement in progression-free survival of crizotinib-treated patients with anaplastic lymphoma kinase (ALK) translocation, metastatic non-small-cell lung cancer (NSCLC) and thereafter changed the treatment of this disease [36, 37]. Although the efficacy of crizotinib is thought to be specific to ALK inhibition, crizotinib also exerts its functions via the generation of O_2_^•−^ and activation of an apoptotic cascade that contributes to cardiomyocyte toxicity in vitro [23]. Accumulation of ROS after crizotinib treatment, somewhat nonspecifically measured by using the general oxidative stress indicator H_2_DCFDA, has been also been reported in human alveolar rhabdomyosarcoma cells [38]. The established cancer stem-cell marker, aldehyde lactate dehydrogenase (ALDH), protected TKI (crizotinib and erlotinib)-resistant cells from TKI-derived ROS toxicity in cell lines [39]. Again, pharmacological disruption of ALDH activity with disulfiram led to accumulation of ROS to toxic levels, consequent DNA damage, and apoptosis. These results have not been tested yet in vivo, but if confirmed, modulation of ROS levels could boost the therapeutic effects of crizotinib.

### 2.3. Erlotinib, Gefitinib, and Afatinib

Gefitinib and erlotinib are TKIs targeted against epidermal growth factor receptor (EGFR) and are approved for the treatment of NSCLC in the first and subsequent lines of therapy. The mechanism of action allows drugs to act only in tumours with activating EGFR mutations, which are found in 10–15% of Caucasian patients. Afatinib is another EGFR-targeted TKI, which has also proven to have activity against T790 M EGFR mutation [40]. Although targeted specifically against EGFR, EGFR-independent mechanisms of action of these drugs have also been reported. Of these three compounds, erlotinib and gefitinib in particular have been linked to oxidative stress in recent literature.

As mentioned above, ALDH-mediated protection against ROS has been connected with erlotinib resistance [39]. In those experiments, erlotinib indeed enhanced ROS production even more considerably than crizotinib. In NSCLC A549 cell lines, erlotinib induced ROS-mediated apoptosis via activation of the c-Jun N-terminal kinase (JNK) pathway, leading ultimately to EGFR inhibition and a therapeutic response. As expected, it was possible to reverse this phenomenon by way of administration of the ROS scavenger N-acetyl cysteine [41, 42].

In recent papers, gefitinib has also been demonstrated to produce a dose-dependent increase in oxidative stress, which has been associated with induced epithelial-mesenchymal transition and cardiotoxicity of this EGFR-targeted TKI [43, 44]. In gefitinib-resistant A549 cells, Prx II was highly upregulated via demethylation of the Prx II gene when compared with the gefitinib-sensitive A549 cell line [45]. Elevated Prx II expression resulted in downregulation of ROS, attenuated apoptosis, increased colony formation, and cell cycle progression in gefitinib-resistant cells, factors that were recovered with Prx II mRNA knockdown. Prx II thus emerges as a potentially targetable factor for overcoming gefitinib resistance. In theory, this may also apply to other EGFR-targeted TKIs, although they have not yet been assessed from this perspective.

Abnormal NFE2L2-Keap1regulation has been associated with the acquisition of resistance to traditional chemotherapy and also to poor prognosis in NSCLC [46, 47]. Recently, NFE2L2 has been characterized as being essentially important in EGFR TKI resistance. In a study by Leone et al. [48], a strong synergistic effect of the histone deacetylate inhibitor vorinostat and EGFR TKIs led to remarkably enhanced antiproliferative and proapoptotic effects of EGFR TKIs, possibly due to underlying changes in REDOX homeostasis. When vorinostat was administered with erlotinib or gefitinib, NFE2L2 levels were notably attenuated via c-Myc downregulation, and simultaneously, Keap1 upregulation was observed.

The EGFR pathway is able to activate NFE2L2 in EGFR wild-type NSCLC after ligand-receptor binding. Also, activation of downstream signalling of the mutated EGFR pathway leads to constitutive expression of NFE2L2. Exposure to oxidative stress in the form of cigarette smoke extract was found to attenuate EGFR-TKI cytotoxicity in EGFR-mutated NSCLC due to oxidative stress-related NFE2L2 activation. Based on their experiments, the authors also hypothesized that inactivating Keap1 mutations could be used as predictive factors of EGFR TKI resistance, which is also supported by very recent results in a paper by Krall et al. [49, 50]. If this could be confirmed in a prospective clinical trial, it would allow more optimal selection of patients for these costly and potentially toxic treatments and development of more specific molecular targets to overcome resistance.

In line with results concerning other EGFR-targeted TKIs in NSCLC, erlotinib also elicits cytotoxicity via oxidative stress generation in head and neck squamous cell carcinoma (HNSCC) cell lines, again being reversible with N-acetyl cysteine [51]. In these experiments, the source of oxidative stress was reported as NADPH oxidase 4-induced H_2_O_2_ production. Afatinib is a less well studied EGFR-targeted TKI, but chronic oxidative stress has been connected to the development of afatinib resistance [52].

### 2.4. Vemurafenib

Vemurafenib was the first BRAF inhibitor to be used in the treatment of inoperable or metastatic melanoma. Despite the overall survival benefit when set against dabrafenib [53], acquired resistance is also a major clinical problem with this drug, and it occurs in virtually all patients sooner or later. Vemurafenib acts by targeting the most common genetic alteration in melanomas, BRAF V600E, and thus it suppresses the RAS/MEK/ERK signalling pathway and accordingly cell proliferation and adhesion. As an additional mechanism of action, vemurafenib stimulates NO^•^ and O_2_^•−^ production and it also induces depolarization of mitochondrial membranes in BRAF V600E-mutated melanoma cells, potentially initiating apoptosis and growth inhibition [54, 55]. Intrinsic high rates of mitochondrial respiration and oxidative stress of vemurafenib-resistant melanomas have been harnessed to overcome resistance to prooxidants, the administration of which leads to notably increased cell death in these already oxidatively stressed cells [56]. On the other hand, in a recent paper by Luo and colleagues [57], vemurafenib was neatly demonstrated to suppress the metastatic potential of melanoma by inducing the oxidative stress regulator PGC1*α* (peroxisome proliferator-activated receptor-gamma coactivator-1*α*) and further suppressing the expression of most integrins. The phenomenon was independent of the cytostatic effect of vemurafenib. All in all, vemurafenib appears to have notable and partly ROS-dependent therapeutic effects, which are independent of BRAF V600E inhibition. As far as we know, no data currently exists about another approved BRAF V600E inhibitor, dabrafenib, and ROS-mediated effects.

### 2.5. Lapatinib

Lapatinib is the only TKI approved for treatment of breast cancer, more specifically its HER2-over-expressing subtype. HER2 is an acquired oncogene that is overexpressed in 20–30% of breast cancer patients. This receptor tyrosine kinase (RTK) drives prosurvival and proliferation signalling, and HER2 expression in breast cancer is associated with aggressive disease and resistance to chemotherapy. Increased ROS levels have been reported after treatment with a lapatinib analogue (GW583340) in inflammatory breast cancer models. In contrast, extremely low ROS levels have been observed in GW583340-resistant models, probably resulting from increased SOD1, SOD2, and GSH expression in lapatinib-resistant breast cancer cells. Elevated antioxidant expression also correlated with decreased lapatinib-analogue efficacy and, most interestingly, from a therapeutic point of view, a SOD mimic was able to overcome resistance in GW583340-sensitive cells [58]. Similarly, Zhang and colleagues [59] demonstrated NFE2L2-mediated ROS suppression in another lapatinib-resistant breast cancer cell line. Also in this work, lapatinib resistance was overcome with ROS level downregulation. In theory, these results could potentially provide a basis for reversing lapatinib resistance by way of ROS level suppressors.

### 2.6. Imatinib

Up to 90% of GISTs harbour activating mutations in platelet-derived growth factor receptor-*α* (PDGFR-*α*) or KIT (CD117) genes. Besides these, imatinib also targets other tyrosine kinases such as ABL and colony-stimulating factor-1 receptor. Imatinib has EMA approval for the treatment of KIT-positive GISTs (in both adjuvant and metastatic settings), inoperable dermatofibrosarcoma protuberans, and chronic myeloid leukaemia (CML). Most of the data relating to the ROS-mediated mechanisms of imatinib are based on leukaemia material, but this is not in the scope of this review. However, auranofin, a gold-containing chemical applied for treatment of rheumatoid arthritis, inhibits thioredoxin reductase, inducing ROS formation and in this way dramatically inhibiting GIST cell growth, and it also induces apoptosis in imatinib-resistant cells [60]. ROS-dependent apoptosis has also been reported in melanoma cell lines after imatinib treatment [61].

## 3. mTOR Inhibitors

Two mammalian target of rapamycin (mTOR) inhibitors, everolimus and temsirolimus, have been approved for treatment of cancer. The oncological indications for everolimus are rapidly widening, and currently, it is used in the treatment of advanced/metastatic breast cancer, RCC, and neuroendocrine carcinomas of lung, pancreatic, or gastrointestinal origin. mTOR inhibitors are also applied in the treatment of graft-versus-host reactions after organ transplants. The available evidence of ROS-mediated effects of mTOR inhibitors is derived solely in connection with everolimus, virtually only concentrating on it as an immunosuppressant, not as an oncological compound. Animal and patient studies are still contradictory in this field. Decreased serum and plasma concentrations of malondialdehyde, protein carbonyls, and oxidized LDL have been reported after both short- and long-term everolimus dosing [62, 63], while after kidney ischaemia/reperfusion injury everolimus was noted to promote both oxidative and nitrosative stress [64]. Thus, based on current information, mTOR inhibitors do not appear to have substantial ROS-mediated roles in the treatment of malignancies, but the field is still understudied.

## 4. Monoclonal Antibodies

### 4.1. Trastuzumab and Pertuzumab

Trastuzumab is a monoclonal antibody that blocks HER2 dimerization with other HER partners, evokes antibody-dependent cellular cytotoxicity, and inhibits MAPK and PI3K/Akt pathways (Vu et al. 2012). Pertuzumab, another humanized monoclonal antibody, inhibits HER2 dimerization with other HER family receptors and significantly enhances the cytotoxicity mediated by trastuzumab in a clinical setting [1]. Trastuzumab is indicated in the (neo) adjuvant therapy of breast cancer and it also has approval in a metastatic setting in HER2-positive gastric and breast cancer. Pertuzumab is indicated in combination with trastuzumab to enhance the anti-HER efficacy, both in neoadjuvant and metastatic settings.

NFE2L2 regulation has a relevant role in RTK signalling and in anti-HER therapies. It has been demonstrated that NFE2L2 positively regulates HER2 and HER3 gene transcription and protein expression, and further, pAKT levels in an ovarian cancer cell line. SiRNA inhibition of NFE2L2 directly inhibited the transcription of HER2 [65, 66]. To further support the crosstalk between these two players, combination treatment with trastuzumab and pertuzumab led to the inhibition of NFE2L2 by promoter methylation. Pharmacological activation of NFE2L2 protected cells from the cytotoxic effect of trastuzumab-pertuzumab treatment and resulted in antioxidant induction. This further emphasizes the notion that NFE2L2 functions as an oncogene and has a major part in evolving drug resistance, even in antibody-based treatment.

Despite the fact that tumour cells and tissue have an increased antioxidant capacity [10], primary and metastatic HER2-positive breast cancer patients have a decreased antioxidant status in their blood, which is restored with trastuzumab combined with chemotherapy up to the levels of healthy controls [67]. On the flip side of (highly effective) trastuzumab treatment, deleterious effects on cardiomyocytes have been widely reported in the literature. Blocking HER2 receptors induces cardiomyocyte death through a mitochondrial pathway that is dependent on ROS and can be reversed by inhibiting Bax and Bac proteins that mediate cell death through this pathway [68].

Just as in any other anticancer therapy, resistance to trastuzumab treatment frequently occurs. One of the putative mechanisms that involves oxidative stress regulation behind trastuzumab resistance is loss of function of the tumour suppressor PTEN due to increased levels of reduced Trx-1 protein. Trx-1 binds to PTEN, enabling full AKT signalling and cell growth, and trastuzumab-resistant cells gained drug sensitivity after treatment with the Trx-1 inhibitor 1-methylpropyl 2-imidazolyl disulphide (PX-12) [69]. Trastuzumab resistance is an immense clinical problem, especially in HER2-positive metastatic breast cancer and in vivo studies with PX-12 to overcome this resistance represent a reasonable step in the future.

Reactive oxygen species also seem to be involved in the regulation HER2 expression after radiation, at least in vitro. Wattenberg and colleagues [70] recently reported that radiation exposure increased cell surface and total protein expression of HER2, EGFR, and CD20 in breast cancer, HNSCC, and non-Hodgkin lymphoma (NHL) cell lines, respectively. HER2 upregulation was mediated for the most part via intracellular production of ROS. Radiation-induced expression led to enhanced antibody-dependent cell-mediated cytotoxicity (ADCC) when breast cancer cells were treated with trastuzumab.

### 4.2. Rituximab

Rituximab is a chimeric humanized monoclonal antibody specific to CD20. CD20 is an integral transmembrane phosphoprotein present on the surface of precursor B-cells, maturing B-cells, and differentiated plasma cells. It is described to be a calcium-channel protein, and binding of rituximab to CD20 causes subsequent calcium influx and downstream apoptotic signalling [71].

Rituximab is indicated in the treatment of NHL and other B-cell malignancies. It sensitizes B-cell lymphoma cells to standard anticancer drugs and restores drug sensitivity of NHL cells via downregulation of antiapoptotic factors, such as Bcl-2, and inhibition of survival signalling of the p38 MAPK signal pathway [72]. In addition, combining irradiation with rituximab to treat a Burkitt's lymphoma cell line was shown to be effective in triggering cell-cycle arrest and apoptosis and bringing about an increase in intracellular ROS levels [72]. Thus, the efficacy of rituximab-combination therapies is often due to the synergy of oxidative stress damage caused by traditional anticancer strategies and the proapoptotic process triggered by rituximab.

In addition to the proapoptotic features of anti-CD20 treatment, rituximab-mediated complement-dependent cytotoxicity involves ROS production, more specifically O_2_^•−^ [73]. Wang et al. studied a highly conserved antiapoptotic molecule, phosphatidylethanolamine-binding protein (hPEBP) and its role in rituximab resistance. It was shown that hPEBP4 was overexpressed in the majority of NHL patients and it inhibited rituximab-mediated complement-dependent cytotoxicity, calcium influx, and ROS generation. Knockdown of hPEBP4 potentiated the chemosensitization effect of rituximab during topoisomerase I inhibitor treatment [74].

In a study by Alinari et al. [75], rituximab was combined with the anti-CD74 antiproliferative agent milatuzumab to test their potential efficacy on mantle cell lymphoma cells isolated from patients. CD74 is a glycoprotein associated with major histocompatibility complex (MHC) class II and functions as an accessory signalling and survival molecule. The study showed that the cytotoxicity of the combination treatment was partially dependent on ROS formation and mitochondrial membrane dysfunction. The results showed that cell death was not due to classic apoptosis nor autophagic mechanisms, and they suggest that cytoskeletal features and antibody capping might be influential factors in increasing the intracellular ROS burst and the loss of mitochondrial membrane polarity. This drug combination was later tested in a phase I/II trial, showing activity in heavily pretreated patients with relapsed or refractory indolent NHL [76].

### 4.3. Bevacizumab

Bevacizumab is a recombinant, humanized monoclonal antibody that targets VEGF, inhibiting ligand binding to VEGFR and ultimately inhibiting neoangiogenesis and vascular leakiness. Bevacizumab is approved for the treatment of CRC and NSCLC, for example. However, the results of antiangiogenic treatment have been disappointing in many cases. Fack and colleagues [77] studied the adaptation of glioblastoma to bevacizumab treatment. Researchers observed that while bevacizumab causes hypoxia in tumours, on the downside, cell metabolism is reprogrammed toward anaerobic metabolism, favouring lactate production. L-cysteine, l-cystathione, and GSH levels were reduced, indicating that oxidative stress levels increased after bevacizumab treatment. This is also expectable in tumours with insufficient vascularization.

Metabolic stress under hypoxia caused by antiangiogenic treatment also upregulates autophagy, which is a means to escape from metabolic stress. There is some evidence that inhibition of autophagy enhances the efficacy of bevacizumab via alteration of the redox balance. In a human HCC study [78], autophagy inhibition led to increased apoptosis and elevated ROS levels during nutrient starvation and hypoxia. Combined treatment with autophagy inhibitor and bevacizumab led to enhanced reduction of xenograft tumour growth. Researchers observed that bevacizumab- and chloroquine-treated cells expressed more 8-hydroxydeoxyguanosine, a marker of DNA oxidative stress damage, than either agent alone. The main conclusion was that autophagy modulates ROS levels, and this could be an efficient way to enhance antiangiogenic treatment. In retinal cells exposed to H_2_O_2_ and bevacizumab, increased bevacizumab concentrations decreased bcl-2 mRNA and increased apoptosis, implying that oxidative stress levels influence the effect of bevacizumab on apoptosis [79].

### 4.4. Cetuximab

EMA-approved indications for cetuximab are CRC and HNSCC. Cetuximab is a recombinant monoclonal antibody designed to target EGFR and is applicable if the downstream signalling pathway does not harbour activating mutations, such as Ras, which are frequent in colorectal cancer. EGFR is overexpressed in 90% of cases of HNSCC.

In addition to MAPK signalling blockade, EGFR inhibition might lead to additional mechanisms that affect cell survival. Lu and colleagues [80] described how cetuximab downregulated a complex in the cell cytoplasmic membrane formed by a glutamine transport protein, ASCT2, and EGFR via endocytosis in HNSCC cell lines. Internalization of glutamine receptor led to decreased glutamine, which is necessary for GSH synthesis, and resulted in decreased ROS-reducing capacity. Increased oxidative stress via this mechanism induced apoptosis independent of EGFR-pathway downregulation. Combined use of oridonine and cetuximab suppressed phosphorylated EGFR formation, increasing ROS and apoptosis in laryngeal carcinoma cells [81].

Conflicting data exists regarding the efficacy of oxaliplatin, one of the most applied chemotherapeutic agents in CRC, and cetuximab, when they are used in combination treatments. Results favouring this combination suggest that cetuximab inhibits DNA repair mechanisms to support oxaliplatin efficacy [82, 83]. In contrast to this, several phase III randomized clinical trials have revealed that despite a survival benefit when cetuximab or oxaliplatin are added to chemotherapy backbones as single agents, their combination does not increase survival [84–86]. An experimental study by Dahan and colleagues [87] showed that cetuximab has an antagonizing effect on oxaliplatin in CRC. The effect of oxaliplatin is dependent on Nox1-evoked ROS formation. When EGFR and the downstream Ras/Nox1 cascade were inhibited by cetuximab, ROS formation decreased and oxaliplatin efficacy was lost. Similar results not favouring the combination were reported by Santoro and colleagues [88]. The efficacy of oxaliplatin in causing apoptosis was ROS-dependent and occurred via signal transducer and activator of transcription 1 (STAT1) and dual oxidase 2 (DUOX2), but cetuximab caused DUOX2 inhibition and p38 activation, reducing the cytotoxicity of oxaliplatin.

## 5. Conclusions

Although new targeted treatments have produced significant leaps ahead in the treatment of solid malignancies and lymphomas, their prolonged use is often limited due to the acquisition of resistance, contributing eventually to treatment failure in metastatic disease. There is a significant clinical need for more accurate predictive factors as regards these drugs, to avoid both clinical and financial toxicity [89].

Based on this review of the literature, monoclonal antibodies and TKIs seem to have many ROS-mediated mechanisms of action, which may be related to their efficacy and also to toxicity. The majority of the reviewed targeted therapy agents increase the oxidative stress burden up to a level that is likely to surpass the reduction capacity of cancerous cells. In this way, they possess antitumour efficacy in addition to their targeted impact. Such agents were anti-VEGFR-PDGFR TKIs (axitinib, pazopanib, and sorafenib), anti-EGFR TKIs (afatinib, erlotinib, and gefitinib), anti-ALK (crizotinib), anti-HER2 TKI (lapatinib), anti-BRAF TKI (vemurafenib), and anti-VEGFR antibody (bevacizumab). They all target cell membrane-related protein structures, RTKs, or associated proteins, with the purpose of conveying external messages into the cell. Not surprisingly, they are also associated with reactive oxygen molecules that can function as second messengers [90].

Some agents such as trastuzumab and pertuzumab inhibit the major redox response regulator NFE2L2. In contrast, increased levels of NFE2L2 and upregulation of other antioxidants lead to tolerance to oxidative stress, a sign that is also related to therapy resistance. Such cases are noted in anti-EGFR and anti-HER2 therapies (trastuzumab, lapatinib), while in the case of vemurafenib, antioxidant upregulation seems to have therapeutic effects. Sunitinib and cetuximab might also have beneficial effects for cancerous tissue via upregulated GSH levels.

On the other hand, as regards many drugs such as the TKIs regorafenib, lenvatinib, and dabrafenib and the monoclonal EGFR inhibitor panitumumab, there is a total lack of evidence of ROS-mediated actions, or these agents remain unstudied from this perspective. The efficacy of the monoclonal anti-CD20 antibody rituximab has been widely studied in non-Hodgkin's lymphoma and leukaemia models in combination with traditional cytotoxic agents and irradiation. Its additive effect is often mediated via the Bcl-2-related mitochondrial apoptotic pathway, enhancing ROS-related cytotoxicity of conventional antitumour therapies [91, 92], but it also increases intracellular oxidative stress and results in a loss of mitochondrial membrane potential.

Rather surprisingly, we did not find any original articles describing ROS-mediated effects of novel oncoimmunological agents such as the CTLA-4 inhibitor ipilimumab or programmed death (PD-1) antibodies such as pembrolizumab or nivolumab. However, a reasonable amount of data exists to point out that an oxidative milieu has an enormous impact on tumour cells, tumour-infiltrating lymphocytes, and other immune cells (and their interactions). It is plausible that these agents have direct ROS-dependent mechanisms arising from interactions between PD-1 antibodies and ROS generation [93]. Since ROS levels and redox status have potential as prognostic or predictive factors of immunotherapy, studies addressing these issues are eagerly awaited.

In conclusion, many TKIs and monoclonal antibodies seem to mediate their effects (and adverse effects) via ROS, and therefore, it is essential to accelerate more systematic studies in this field. In addition, we should further study the potential antagonizing effects of targeted therapies when they are combined with traditional chemotherapeutic agents, as discussed in the context of cetuximab.

## Figures and Tables

**Table 1:** The most important redox-associated targeted cancer therapy compounds with EMA-approved indications for the treatment of solid tumours or lymphomas. All indications are for metastatic or inoperable carcinomas if not otherwise mentioned.

Drug	EMA-approved indication in solid tumours	Main targets	Role in redox system
Afatinib	EGFR-mutated NSCLC	EGFR	Chronic oxidative stress associated with resistance.

Axitinib	RCC	VEGFR1–3, PDGFR, c-Kit	Oxidative stress-mediated genotoxic effects.

Bevacizumab	CRC Breast cancer NSCLC Ovarian cancer Peritoneal cancer RCC	VEGF	Increases ROS levels. Combined with autophagy inhibitor enhances ROS levels and apoptosis.

Cetuximab	HNSCC CRC	EGFR	Reduces the amount of GSH by internalizing EGFR and glutamine transport. Decreases Nox1 and Nox1-related effect of oxaliplatin.

Crizotinib	ALK-positive NSCLC	ALK, c-MET	Increased O_2_^•−^ production linked with cardiotoxicity. Prx II up-regulation associated with resistance.

Erlotinib	EGFR-mutated NSCLC Pancreatic cancer	EGFR	Increases ROS-mediated apoptosis in HNSCC and NSCLC.

Gefitinib	EGFR-mutated NSCLC	EGFR	Increases oxidative stress linked to EMT and cardiotoxicity. NFE2L2/Keap1-axis related to treatment resistance.

Imatinib	GISTs	PDGFR*α*, KIT, ABL, CSF-1 receptor	Induces ROS-dependent apoptosis in melanoma.

Lapatinib	HER2-positive breast cancer	HER1, HER2	Increases ROS; low ROS levels linked with resistance, which may be overcome with antioxidant mimics.

Pazopanib	RCC, sarcomas	Various kinases, for example, VEGFR1–3, (PDGFR-*α* and PDGFR-*β*), c-Kit, FGFR-1, and FGFR-3	May induce oxidative DNA damage-mediated erythrocyte apoptosis.

Rituximab	Non-Hodgkin's lymphoma	CD-20	CD20 stimulation leads to the production of O_2_^•−^.

Sorafenib	HCC, RCC, radioiodine-refractory thyroid cancer	Various kinases, for example, VEGFR-2 and VEGFR-3, PDGFR-*β*, and RAF-kinases	Increases oxidative stress, which possibly is a predictive factor for sorafenib

Sunitinib	GISTs, pancreatic NET, RCC	Various kinases, for example, VEGFR1–3, (PDGFR-*α* and PDGFR-*β*), c-Kit	Enhances antioxidant defence, decreases NOS activity and expression.

Trastuzumab	HER2-positive breast cancer and HER2-positive gastric cancer	HER2 dimerization	Regulatory loop with *NFE2L2* NFE2L2 increases trastuzumab resistance.

Vemurafenib	BRAF (V600E) mutated melanoma	BRAF V600E	Increases NO^•^ and O_2_^•−^ production increases depolarization of mitochondrial membranes. Induces PGC1*α*
